# Issue Information

**DOI:** 10.1002/hsr2.72

**Published:** 2019-11-21

## Abstract

No abstract is available for this article.

